# British Institutions for the Care of the Inebriate—V

**Published:** 1903-11-28

**Authors:** 


### BRITISH INSTITUTIONS FOR THE CARE OF THE INEBRIATE.—V.

By Our Special Medical Commissioner.

(*Continued from Page* 138.)

### QUEENSBERRY LODGE, HOLYROOD, EDINBURGH.

The ever-delightful Robert Louis Stevenson, in his "Picturesque Edinburgh," has portrayed in living language the fascinations of "Auld Reekie," and even the casual visitor to the ancient portions of Scotland's capital is captivated by the quaint and curious, although it must be admitted, frequently repelled by the noise and squalor and sickened by the sight of human degradation and self-inflicted misery. Queensberry House, 64 Canongate, once revelled in the grandeur of high state, but its dignity has decayed, the night of destitution has fallen and little but the ancient residence remains to indicate the glories that have passed.

Queensberry House is now "A House of Refuge for the Destitute." It was instituted in 1832, and is conducted by a Board of Directors, the president being the Lord Provost of Edinburgh. The late Dr. G. W. Balfour, until his death, acted as consulting physician. Dr. William Russell is the visiting physician. The house governor and treasurer is Major D. McCartney. The institution provides a general refuge for persons in a state of complete temporary destitution, persons of intemperate habits boarded by their friends for the purpose of protection and reformation, cases suffering from incurable diseases, and army and navy pensioners. There is an ever-open night refuge. The soup kitchen provides a quart of excellent soup and a 6 oz. roll of bread for one penny, and both are of excellent quality, as we have had an opportunity of testing. Free meals are provided for neglected children.

The directors of Queensberry House long since realised the necessity of providing accommodation for persons of the better class who were the subjects of inebriety, and in 1866, within their own grounds, provided a separate building which they named Queensberry Lodge. This establishment has a separate access close to Holyrood Palace, with a pleasant outlook towards Arthur's Seat and the Queen's Park. The Lodge is arranged to accommodate about 30 residents. Between August, 1866, and September, 1902, 1,059 cases have been admitted, and of these 775 came from Scotland, 177 from England, 103 from Ireland, and 4 from Canada. Four classes of boarders are taken. The so-called first class have a sitting-room and bedroom, for which £100 per annum is the nominal charge. The second-class boarders have a bedroom-sitting-room, and pay £80 per year. The third-class boarders have a room answering also as a bedroom, and the charge is £60; while the fourth-class boarders share a room with another patient, each paying £50. Fire in a private room is charged 10s. extra per month, and washing is 6s. per month for all boarders. The dietary, we understand, is "in correspondence with rate of board." The first and second-class boarders have their meals served in their own rooms, while other boarders take their meals together. Among the regulations we observed that "The board must be paid in advance. No money taken under one month, and no money returned though the party leave before the expiry of the time paid for."

At the time of our visit we found several of the rooms occupied by infirm, invalid, and senile cases. We are of opinion that the Board of Management would be well advised in modernising their establishment and bringing their methods into accord with the best recent procedures for conducting the restoration of the inebriate. Queensberry Lodge might with advantage be removed into the country, where full advantage of a hygienic régime might be afforded the patients, and where treatment might be conducted on the now approved sanatorium lines. We consider it undesirable to provide for other classes of patients than inebriates in the same building. Queensberry Lodge as at present conducted is too much "a home for invalid and aged ladies," and too little "a temperance home for ladies." It is very desirable that it should be converted into a properly-licensed retreat for inebriates where constant medical supervision might be available and the best possible environment provided for this most unfortunate class of sufferers.

The researches of Dr. Clouston and the recent investigations of Mr. Arthur Sherwell have clearly indicated the growing addiction of women in Scotland to excessive alcoholic indulgence, and from our own observations we are convinced of the widespread prevalence of intemperance in Edinburgh. We therefore think the Board of Management of Queensberry Lodge have not only a heavy responsibility but a great opportunity, and should provide a thoroughly efficient, modern and scientifically conducted establishment for female inebriates in Edinburgh and district where rational treatment in accordance with the best up-to-date methods may be secured.

(*To be concluded.*)

